# Age-stratified serosurvey reveals immunity patterns against measles in Youyang, China

**DOI:** 10.3389/fpubh.2026.1888095

**Published:** 2026-07-21

**Authors:** Siyu Chen, Tianlan Wang, Kaiwei Liu, Qing Wang, Wei Shi, Xiaoping Cheng, Kaihu Yao

**Affiliations:** 1 Beijing Key Laboratory of Core Technologies for the Prevention and Treatment of Emerging Infectious Diseases in Children, Key Laboratory of Major Diseases in Children, Ministry of Education, National Key Discipline of Pediatrics, National Clinical Center for Pediatric Infectious and Allergic Disease Surveillance, National Clinical Research Center for Respiratory Diseases, Laboratory of Infection and Microbiology, Beijing Pediatric Research Institute, Beijing Children’s Hospital, Capital Medical University, National Center for Children’s Health, Beijing, China; 2 Clinical Laboratory, Youyang Hospital, A Branch of The First Affiliated Hospital of Chongqing Medical University, Chongqing, China

**Keywords:** ELISA, immunity, measles, seroprevalence, vaccination

## Abstract

**Objective:**

To evaluate age-specific measles antibody dynamics and identify population immunity gaps in Youyang County, China, approximately two decades after the introduction of a two-dose measles-containing vaccine (MCV) schedule at 8 and 18 months of age.

**Methods:**

A cross-sectional serosurvey was conducted among residents without rash in 2025. Measles-specific IgG antibodies were quantified using enzyme-linked immunosorbent assay (ELISA), with seroprevalence defined as >200 mIU/mL. Seroprevalence and geometric mean concentrations (GMCs) were analyzed across eight age strata.

**Results:**

Among 608 participants, the crude overall seroprevalence was 76.5% (IPW-adjusted rate: 81.5%), with significant age-specific variation (*p* < 0.001). The lowest levels were observed in infants aged <8 months (36.5, 95%CI: 26.2–47.9; GMC: 120.47 mIU/mL). Following two vaccine doses, peak immunity was achieved in children aged 18 months to <6 years (>95%; GMC: 730.99–1044.61 mIU/mL). Seroprevalence then reached a distinct nadir in adults aged 18–<40 years (65.6, 95% CI: 50.9–77.9; GMC: 330.48 mIU/mL), before rebounding in older adults aged ≥40 years (90.1–93.5%).

**Conclusion:**

This investigation revealed a distinct immunity gap, with measles adjusted seroprevalence at 36.5% (95% CI: 26.2–47.9) in infants under 8 months and 65.6% (95% CI: 50.9–77.9) in reproductive-aged adults, despite robust post-vaccination responses in young children. This serological pattern may be a common characteristic in populations that complete two-dose MCV immunization by 18 months of age and experience persistently low measles incidence.

## 1 Introduction

Measles poses a severe threat as a respiratory disease with intense contagiousness (1). Vaccination is among the most cost-effective strategies to control and eliminate measles, dramatically slashing its incidence and transmission scale by building population immunity (2). In 2000, the United States became the first country to declare the elimination of measles (3). Later, the World Health Assembly adopted the Global Vaccine Action Plan (GVAP) in 2012, aiming to achieve measles elimination in no fewer than five of the six World Health Organization (WHO) regions by 2020 (4). In 2020, to consolidate and accelerate global progress, WHO unveiled the “Measles and Rubella Strategic Framework 2021–2030” (5). With a vision of “a world without measles and rubella,” this framework was designed to steer more effective national and regional elimination efforts. Subsequently, global measles vaccination coverage plummeted to its lowest level since 2008 due to the suspension of immunization services, during the COVID-19 pandemic. Lack of measles vaccination led to a resurgence of the disease worldwide (2), as evident by a doubling of reported cases in the European Region in 2024 compared to 2023 (6), and a nearly five-fold increase in the U.S. which may be attributed to increased importation via travel alongside gaps in vaccination and waning immunity. Explosive local transmission has been observed there since the beginning of 2025 (7). Concurrently, other countries in the WHO Western Pacific Region, such as Japan and the Republic of Korea, have also documented significant measles outbreaks in recent years (8, 9).

In China, a single-dose measles vaccination schedule for infants at 8 months of age was initiated nationwide in 1978. In 1986, this was transitioned to a two-dose regimen, with the first dose given at 8 months and the second dose at 7 years of age. The schedule was further optimized in 2005, when the administration of the second dose was advanced to 18 months of age. A significant update in 2008 introduced combination vaccines: the measles-rubella (MR) vaccine at 8 months and the measles-mumps-rubella (MMR) vaccine at 18–24 months. In June 2020, China’s immunization program was further unified by transitioning to the MMR vaccine for both doses nationwide. The coverage rate of measles-containing vaccines (MCV) among the target pediatric population has remained exceptionally high, where reported rates exceeding 99% from 2013 to 2018 (10). Despite a brief initial impact from the COVID-19 pandemic in early 2020, the coverage of MMR vaccine among Chinese children remained consistently above 97% during 2022–2024 (11).

As a consequence of active measles vaccination, from 2010 to 2019 the national measles incidence has fluctuated between 2 and 4 per 100,000. In 2020, China reported a historic low measles incidence of 0.06 per 100,000 population (856 cases in total). Nevertheless, domestic transmission has persisted. However, since 2022, the number of reported measles cases in China has shown a progressive increase in recent years, rising from 552 in 2022, to 621 cases in 2023, and a marked surge to 1,458 cases in 2024 and 1,864 in 2025 (12). Although the incidence rate remains relatively low, this rise in cases suggests a potential shift in population susceptibility.

Measles immunity acquired through vaccination or natural infection is known to wane progressively with age (13). The persistently low incidence of measles has also reduced opportunities for naturally enhanced immunity. In the context of childhood-only immunization programs, the immunization levels of the overall population may undergo substantial changes over time. Crucially, this shifting epidemiological landscape fundamentally alters the dynamics of passive maternal immunity. In highly vaccinated populations, women of childbearing age increasingly possess vaccine-induced immunity rather than immunity from natural infection. Because vaccine-induced maternal antibodies typically exhibit lower baseline titers and faster clearance kinetics, infants are prematurely exposed to a critical window of susceptibility long before their first scheduled vaccine dose at 8 months (13, 14). Therefore, identifying the distribution of susceptible populations is essential to provide an evidence-based foundation for adjusting and optimizing immunization strategies. Accordingly, this study was designed to analyze the measles seroprevalence across all age groups in Youyang County, where the national childhood measles immunization program has been strictly and consistently implemented since the administration of MCV in China. It was anticipated that the findings would provide useful insights for optimizing measles prevention and control strategies.

## 2 Materials and methods

### 2.1 Participants and age group

This study was conducted among the local resident population seeking care at the Youyang County People’s Hospital in Chongqing. The hospital primarily serves a population of approximately 250,000 individuals from both the main urban areas and surrounding suburbs. A consecutive convenience sampling strategy was employed in this study. Participants were recruited from patients attending the Youyang County People’s Hospital between March and May 2025, including both inpatient and outpatient settings. During this three-month window, residual serum samples from all consecutive individuals who met the inclusion criteria were collected, only a single residual serum sample per individual was included. We did not perform an *a priori* sample size calculation, nor was the sampling stratified based on the local demographic age distribution. Instead, the total sample size and the varying sample sizes across different age groups were naturally dictated by the demographic flow of hospital attendees during the study period. Individuals with current rash, fever, chronic infectious diseases, immunosuppressive conditions, a documented history of receiving intravenous immunoglobulin (IVIG), plasma, or whole blood transfusions within the 6 months prior to sample collection were excluded from the study. Basic demographic information, including age or date of birth and sex, was also recorded.

**Figure 1:**
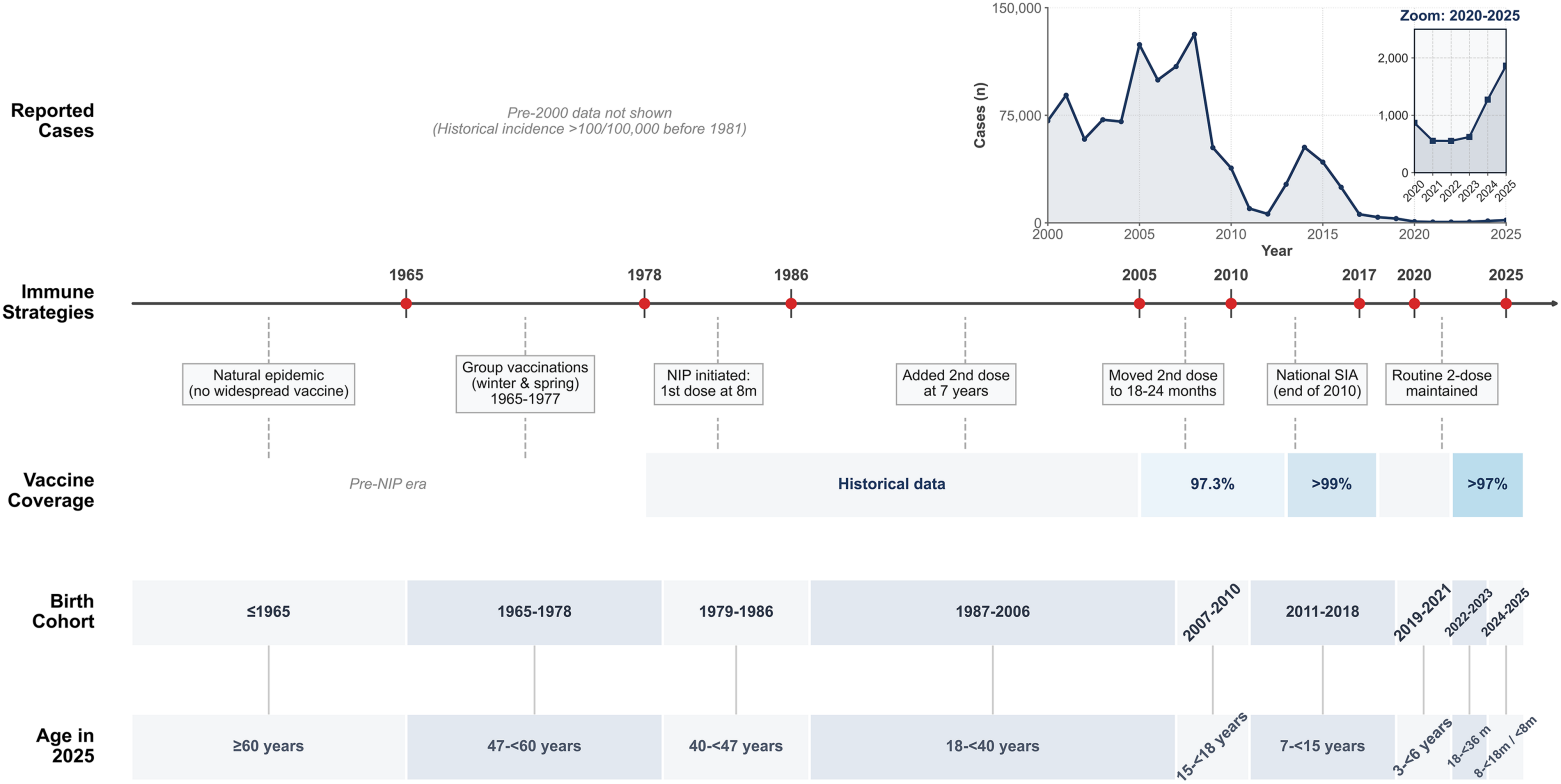
Evolution of measles immunization strategies and corresponding birth cohorts with age in 2025, China. Data for reported cases were obtained from the World Health Organization (6).

Because medical records in Chinese hospitals are not linked to immunization information systems, this study lacked access to verified individual-level MCV vaccination data. However, as the timely vaccination rate for measles vaccines has consistently served as an important indicator for evaluating vaccination status in China, it can be assumed that all respondents received the vaccine on schedule after the introduction of MCV. Based on the historical changes in the measles vaccine and its immunization program in Chinese mainland as described in the introduction and shown in Figure 1, and in accordance with conventional age classification methods for children, adolescents, and other age groups, this study divided the participants into eight age groups: <8 months, 8- < 18 months, 18- < 36 months, 3- < 6 years, 6- < 18 years, 18- < 40 years, 40- < 60 years, and ≥60 years. Notably, the 6- < 18 years and 40- < 60 years groups were each further subdivided into two subgroups (6- < 15 years and 15- < 18 years, and 40- < 47 years and 47- < 60 years, respectively) to explore the potential effects of key changes in immunization strategies. Individuals born before 1978 (aged ≥47 years in 2025) were considered unvaccinated, whereas those aged 15–18 years represented the first cohort to complete two doses of measles vaccine by 18 months of age and to participate in the last national supplementary immunization activity at the end of 2010.

### 2.2 Sample collection and serological testing

Serum samples were stored at −80 °C and shipped in batches to a laboratory in Beijing for analysis. All testing was completed within 6 months of sample collection. The anti-Measles IgG antibodies were quantitatively detected using the SERION ELISA classic anti-Measles Virus IgG kit (Germany), strictly in accordance with the manufacturer’s instructions. According to the manufacturer, the diagnostic sensitivity and specificity of the assay are >99.0 and 95.0%, respectively. Antibody titers were reported in mIU/mL. The lower and upper detection limits of the assay were 50 mIU/mL and 5,000 mIU/mL, respectively. According to the manufacturer’s instruction, IgG levels >200 mIU/mL were considered positive. Equivocal IgG levels (150–200 mIU/mL) and negative levels (<150 mIU/mL) were grouped together and conservatively handled as negative for the purpose of calculating seroprevalence.

### 2.3 Statistical analysis

Statistical analysis was performed using SPSS 26.0. Antibody concentrations below the lower detection limit (50 mIU/mL) were assigned a value of 25 mIU/mL (half of the threshold). Categorical data were presented as counts (n) and percentages (%). Antibody levels were summarized using the 25th, 50th (median), and 75th percentiles, as well as the geometric mean concentration (GMC). Seroprevalence was reported with 95% confidence intervals (CI) calculated using the Clopper-Pearson exact method. To adjust for the convenience sampling design and improve epidemiological interpretability, post-sample adjustment was performed using the inverse probability weighting (IPW) method using the raw data. Individual sampling weights for each age-and-sex stratum were calculated as the ratio of the target population proportion (derived from the 2020 Chinese National Census) to the observed sample proportion (15). These IPW weights were applied to individual-level data to estimate the adjusted overall seroprevalence. A detailed comparison between our sample demographics and the census data, including the weights used for standardization, is provided in Supplementary Table S1. Furthermore, the Rogan-Gladen estimator was applied to calculate the adjusted seroprevalence estimate based on the assay’s sensitivity and specificity (16). The *χ*^2^ test and Fisher’s exact test were used for significance comparison between groups. Cochran-Armitage trend test was used for trend analysis. Two-sided tests were applied to the data, using a significance level of 5% in each test.

## 3 Results

### 3.1 Characteristics and demographics

A total of 608 serum samples were collected, including 344 (56.6%) males and 264 (43.4%) females. The distribution of participants across age groups is summarized below, and these data are also clearly presented in Table 1: <8 months (14.6%), 8- < 18 months (11.5%), 18- < 36 months (9.5%), 3- < 6 years (8.6%), 6- < 18 years (20.1%), 18- < 40 years (8.9%), 40- < 60 years (17.6%), and ≥60 years (9.2%).

**Table 1:** Measles crude and adjusted seroprevalence estimates (in percentage) and antibody levels by age group in Youyang, 2025.

Age group^a^	Birth	*N* (%)	Crude seroprevalence (95% CI)	Adjusted seroprevalence^b^ (95% CI)	Antibody concentration (mIU/mL)	
					Median (P25-P75)^c^	GMC^d^
<8 m	2024–2025	89 (14.6)	39.3% (29.1–50.3)	36.5% (26.2–47.9)	154.17 (33.67–324.15)	120.47
8- < 18 m	2024–2025	70 (11.5)	72.9% (60.9–82.8)	72.2% (60.0–82.3)	823.82 (85.34–1202.31)	389.21
18- < 36 m	2022–2023	58 (9.5)	96.6% (88.1–99.6)	97.4% (88.9–100.0)	1049.91 (824.60–1549.54)	1044.61
3- < 6 y	2019–2021	52 (8.6)	96.2% (86.8–99.5)	97.0% (87.4–100.0)	853.69 (626.00–1039.03)	730.99
6- < 18 y	2007–2018	122 (20.1)	73.0% (64.2–80.6)	72.3% (63.2–80.4)	305.84 (187.00–584.61)	287.51
6- < 15 y	2011–2018	81 (13.3)	77.8% (67.2–86.3)	77.4% (66.5–86.4)	361.10 (207.94–619.42)	344.33
15- < 18 y	2007–2010	41 (6.7)	63.4% (46.9–77.9)	62.1% (45.2–77.0)	241.86 (127.73–356.97)	189.98
18- < 40 y	1985–2006	54 (8.9)	66.7% (52.5–78.9)	65.6% (50.9–77.9)	316.38 (179.94–591.02)	330.48
40- < 60 y	1965–1986	107 (17.6)	89.7% (82.3–94.8)	90.1% (82.5–95.3)	560.43 (335.90–886.06)	523.33
40- < 47 y	1979–1986	33 (5.4)	81.8% (64.5–93.0)	81.7% (64.4–92.3)	480.98 (235.80–975.58)	484.52
47- < 60 y	1965–1978	74 (12.2)	93.2% (84.9–97.8)	93.9% (85.5–98.2)	580.86 (354.22–855.33)	541.62
≥60 y	≤1965	56 (9.2)	92.9% (82.7–98.0)	93.5% (83.1–98.4)	948.86 (563.89–1336.16)	810.10
Total	—	608(100.0)	76.5% (72.9–79.8)	76.0% (72.3–79.5)	522.52 (211.53–955.08)	395.82

a. m, months; y, years. b. Adjusted seroprevalence estimated based on ELISA diagnostic sensitivity (> 99.0%) and specificity (95.0%) using the Rogan-Gladen estimator. Note: The total adjusted seroprevalence here (76.0%) is based on the crude sample, whereas the population-level estimate after further IPW weighting (81.5%) is discussed in the text. c. P25 and P75 represent the 25th and 75th percentiles, respectively. d. GMC: geometric mean concentration.

### 3.2 Characterization of measles IgG antibodies

The crude overall measles-specific IgG seroprevalence was 76.5% (95% CI: 72.9–79.8). To adjust for sampling bias, we calculated the IPW-adjusted overall seroprevalence as 81.5% based on the 2020 Chinese National Census (Supplementary Table S1). Furthermore, after adjusting for the diagnostic sensitivity (99.0%) and specificity (95.0%) of the ELISA kit using the Rogan-Gladen estimator, the adjusted population seroprevalence was estimated to be 81.3%. No significant difference between the seroprevalence of males and females was observed (76.7, 95% CI: 71.8–81.0 vs. 76.1, 95% CI: 70.4–81.1; *p* = 0.468).

**Figure 2:**
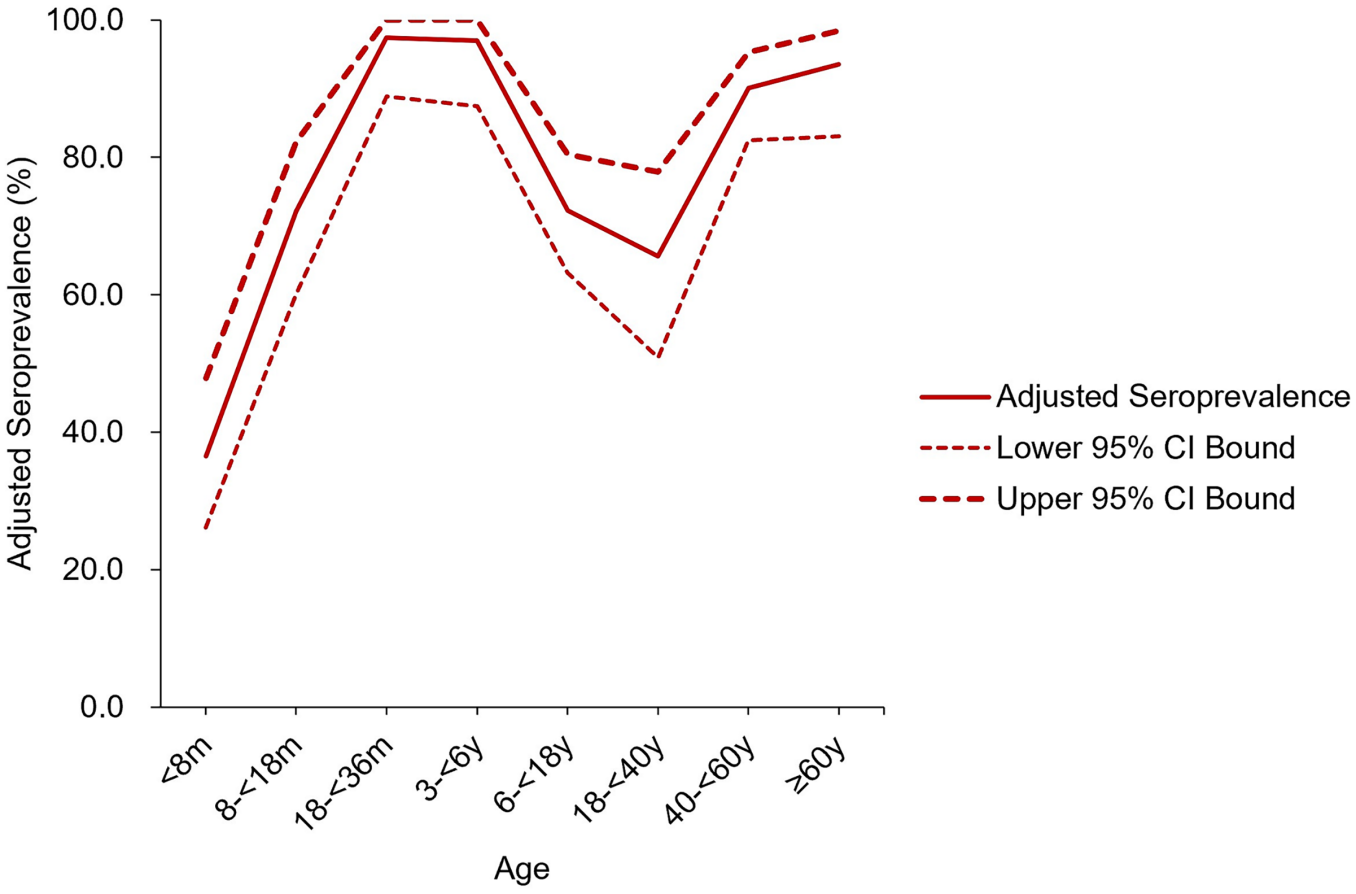
Adjusted seroprevalence (%) of anti-measles IgG antibodies of different age groups.

The seroprevalence exhibited a distinct age-specific distribution (*p* < 0.001), with temporal trends further illustrated in Figure 2. Infants <8 months demonstrated the lowest seroprevalence (36.5, 95% CI: 26.2–47.9) in all groups. Trend analysis revealed that following the peak induced by the two-dose vaccination, the seroprevalence demonstrated a significant decreasing trend from the 3- < 6 years group to the 18- < 40 years group (*P_trend_* < 0.001). Conversely, from the young adult nadir (18- < 40 years) onward, the seroprevalence showed a significant increasing trend with advancing age into middle and older adulthood (up to ≥60 years) (*P_trend_* < 0.001).

### 3.3 Changes in antibody concentrations

Antibody concentrations, evaluated using both median and GMC, revealed age-related trends consistent with seroprevalence (Table 1). The GMC of measles antibodies was lowest in infants under 8 months of age at 120.47 mIU/mL. After the first dose, the GMC increased by approximately three times, reaching 389.21 mIU/mL in the 8- < 18 months age group. The highest level was observed in children aged 18 months to <3 years who had received two doses of immunization, at 1044.61 mIU/mL. The GMC then decreased to 730.99 mIU/mL in those aged 3- < 6 years and continued to decline to 189.98 mIU/mL in those aged 15- < 18 years. Thereafter, antibody GMC levels increased slowly with age, rising to 330.48 mIU/mL in the 18- < 40 years age group, and eventually reaching 810.10 mIU/mL in individuals aged 60 years and older.

## 4 Discussion

It is well-known that the transmission of measles within a population can be interrupted when the seroprevalence surpasses a specific herd immunity threshold (17). This study did not perform sampling based on the overall age distribution of the local population. Therefore, the overall measles antibody level reported here may not accurately reflect the actual seroprevalence in the general population. However, the present results clearly demonstrate two distinct age groups with insufficient immunity have emerged within the Chinese population (<8 m and 18- < 40y age group), as evidenced by both seroprevalence and antibody concentration levels.

The first population that warrants particular attention is unvaccinated infants under 8 months of age, who exhibit the lowest measles seroprevalence (36.5, 95% CI: 26.2–47.9) and the lowest antibody concentration (120.47 mIU/mL) among all age groups. Similar seroprevalence were recently reported from other regions in China: a study in Shanghai (2018–2022) reported a rate of 31.82%, while another in Nantong (2019–2023) documented 27.42% (18, 19). These results collectively demonstrate that infants who have not yet reached the age for their first measles vaccination are not afforded adequate protection by maternal antibodies. The present results reveal that the scheduled first MCV dose at 8 months of age led to a significant rise in both measles seroprevalence (72.2, 95% CI: 60.0–82.3) and GMC (389.21 mIU/mL) in those aged 8- < 18 months. Generally, although delaying the administration of the first vaccine dose may increase the risk of exposure for infants prior to vaccination, it can enhance the immunogenicity (i.e., effectiveness) of the initial dose (1). During the early application of the measles vaccine in the United States (1963), the recommended age for the first dose was 9 months. This was subsequently adjusted to 12 months in 1965 and 15 months in 1976, precisely based on research evidence demonstrating superior immune responses achieved at older vaccination ages (3). In contrast to the United States, Bokop et al., based on findings from African regions, reported that 49.9% of newborns tested negative for serum measles antibodies, placing them at an extremely high risk of infection immediately after birth. Consequently, they recommended advancing the first dose of the measles vaccine in African regions from the WHO-recommended 9 months to 4–6 months of age (20).

In China, the schedule for the first dose of the measles vaccine was set at 8 months of age for over four decades. Currently, the reported incidence of measles in China is gradually approaching and reaching the elimination level, accompanied by consistently high childhood vaccination coverage and no indications of large-scale epidemics (2). In this context, given the very low antibody levels in infants under 8 months of age, whether and how to adjust the timing of the first vaccine dose requires detailed investigation and research into epidemic trends and the dynamic changes in infant antibody levels after birth.

It is noteworthy that the adjusted seroprevalence of 72.2% (95%CI: 60.0–82.3) observed in the 8- < 18 months group following the first MCV dose is lower than the 85–95% seroprevalence typically reported in clinical literature (1). Several factors may contribute to this discrepancy. Without linked individual vaccination records, some infants in this age bracket may have been sampled shortly after reaching 8 months of age but before actual vaccination, or before the 2 to 4 weeks needed to develop a mature antibody response. In addition, hospital-based sampling variability and programmatic factors, such as minor vaccination delays or potential cold chain vulnerabilities in rural settings where access to healthcare is limited, cannot be entirely ruled out. Importantly, despite the lower-than-expected response after the first dose, administration of the second dose successfully restored immunity to optimal levels. Adjusted seroprevalence reached 97.4% (95%CI: 88.9–100.0) in children aged 18 months to <3 years and remained high at 97.0% (95%CI: 87.4–100.0) in those aged 3 to <6 years. These data strongly support the current recommendation of a two dose MCV schedule for children (1).

The second population requiring attention revealed is the reproductive age group, in whom the second nadir in seroprevalence and concentration precisely occurs. Although a lower measles seroprevalence and concentration were observed among a sub-group of individuals aged 15- < 18 years (62.1, 95% CI: 45.2–77.0 and 189.98 mIU/mL), the second nadir across the age spectrum primarily occurred in the 18- < 40 years age group (65.6, 95% CI: 50.9–77.9 and 330.48 mIU/mL).

While vaccine-induced antibodies naturally wane over time, natural measles infection generally confers stronger, lifelong immunity (21). However, the age-related serological patterns observed in our study are not solely a result of physiological antibody waning. Rather, they likely reflect a combination of cohort effects, historical shifts in immunization policies, varying levels of natural infection exposure, and differential vaccination uptake. For instance, the high immunity maintained in adults aged ≥40 years (particularly those born before 1978) can be primarily attributed to widespread natural infection prior to the routine use of the measles vaccine. In contrast, the lower antibody levels in the 15- < 40 years age groups highlight specific cohort effects tied to China’s evolving immunization strategies. These individuals were born during transitional periods of the national program, such as the shift from a single to a two-dose schedule, and experienced varying coverage during supplementary immunization activities like the one in 2010 (22). Historical differential vaccination uptake during these transitions may have left gaps in immunity. Furthermore, the sustained low incidence of measles in China provides almost no opportunities for natural immune boosting. Consequently, these cohorts rely almost entirely on early childhood vaccination. Consequently, their current lower antibody levels are the combined result of initial vaccination gaps, the lack of natural re-exposure, and the progressive waning of vaccine-induced antibodies.

This study has several important limitations. First, due to the use of de-identified residual clinical samples from a single hospital (including both inpatient and outpatient settings), detailed clinical histories such as prior natural measles infection and primary reasons for hospital attendance were completely inaccessible. This introduces substantial selection bias, as the immunity profile of individuals seeking medical care may differ from the healthy general population, and we could not adjust for prior infection as a confounder. Second, while the county strictly implements the National Immunization Program, exact historical coverage could not be retrospectively documented to correlate with our serological findings. Correlating seroprevalence with local incidence data would significantly strengthen our conclusions. Unfortunately, granular, age-stratified yearly incidence data for the specific catchment area (Youyang County) is not publicly available due to local health data privacy and reporting regulations. Third, the lack of individual-level vaccination records and our cross-sectional design preclude establishing strict causal relationships and limit our understanding of longitudinal antibody dynamics. Finally, immunity was assessed solely by IgG ELISA, which has relatively low sensitivity at low antibody concentrations compared to the plaque reduction neutralization test (PRNT). Despite these limitations, the serological characteristics revealed here align closely with broader epidemiological trends in China and underscore critical gaps in passive maternal immunity and young adult protection. Future longitudinal, multicenter studies employing more sensitive methods will provide a more definitive assessment of measles immunity dynamics to guide precision immunization policies.

## 5 Conclusion

Based on the serological data in Youyang County, this study highlights low measles immunity in two specific age groups: infants under 8 months of age and adults aged 18- < 40 years. While the current two-dose schedule achieves high seroprevalence in young children, antibody levels decline after 3 years of age and form a trough in reproductive aged adults. This decline poses a potential risk for future resurgence and threatens unprotected young infants who have not yet been vaccinated. This serological pattern may be a common characteristic in populations that complete two-dose MCV immunization by 18 months of age and experience persistently low measles incidence. However, the cross-sectional findings alone cannot determine whether national immunization schedules should be altered. Therefore, China urgently requires larger scale serological surveys across the population to accurately assess measles immunity status and inform evidence-based immunization decisions.

## Data Availability

The raw data supporting the conclusions of this article will be made available by the authors, without undue reservation.
