# Supplementary material for: Age-stratified serosurvey reveals immunity patterns against measles in Youyang, China
Source: Front Public Health. 2026 Jul 21;14:1888095. doi: 10.3389/fpubh.2026.1888095 (PMC13433429; doi:10.3389/fpubh.2026.1888095)
Supplement: Supplementary file 1 [file Table_1.DOCX]

Supplementary Material

# 1 Supplementary Table S1

Supplementary Table S1. Demographic comparison between the study sample and the 2020 Chinese National Census population.

| Demographic Variable | Study Sample (N = 608) | Sample Proportion (%) | Sample Seropositive (n) | Sample Seroprevalence (%) | 2020 Chinese Census Proportion (%) | IPW Weight (Wi) | Adjusted Contribution (%) |
| --- | --- | --- | --- | --- | --- | --- | --- |
| Sex |  |  |  |  |  |  |  |
| Male | 344 | 56.58% | 264 | 76.74% | 51.24% | - | - |
| Female | 264 | 43.42% | 201 | 76.14% | 48.76% | - | - |
| Age Group & Sex |  |  |  |  |  |  |  |
| <8 months | 89 | 14.64% | 35 | 39.33% | 0.57% | - | - |
| Male | 53 | 8.72% | 23 | 43.40% | 0.30% | 0.0344 | 0.13% |
| Female | 36 | 5.92% | 12 | 33.33% | 0.27% | 0.0456 | 0.09% |
| 8-<18 months | 70 | 11.51% | 51 | 72.86% | 0.71% | - | - |
| Male | 49 | 8.06% | 35 | 71.43% | 0.38% | 0.0471 | 0.27% |
| Female | 21 | 3.45% | 16 | 76.19% | 0.33% | 0.0957 | 0.25% |
| 18-<36 months | 58 | 9.54% | 56 | 96.55% | 1.35% | - | - |
| Male | 37 | 6.09% | 36 | 97.30% | 0.72% | 0.1182 | 0.70% |
| Female | 21 | 3.45% | 20 | 95.24% | 0.63% | 0.1826 | 0.60% |
| 3-<6 years | 52 | 8.55% | 50 | 96.15% | 3.00% | - | - |
| Male | 26 | 4.28% | 25 | 96.15% | 1.60% | 0.3738 | 1.54% |
| Female | 26 | 4.28% | 25 | 96.15% | 1.40% | 0.3271 | 1.35% |
| 6-<18 years | 122 | 20.07% | 89 | 72.95% | 13.50% | - | - |
| Male | 79 | 12.99% | 58 | 73.42% | 7.20% | 0.5543 | 5.29% |
| Female | 43 | 7.07% | 31 | 72.09% | 6.30% | 0.8911 | 4.54% |
| 18-<40 years | 54 | 8.88% | 36 | 66.67% | 29.00% | - | - |
| Male | 13 | 2.14% | 9 | 69.23% | 15.10% | 7.0561 | 10.45% |
| Female | 41 | 6.74% | 27 | 65.85% | 13.90% | 2.0623 | 9.15% |
| 40-<60 years | 107 | 17.60% | 96 | 89.72% | 33.17% | - | - |
| Male | 55 | 9.05% | 48 | 87.27% | 16.82% | 1.8586 | 14.68% |
| Female | 52 | 8.55% | 48 | 92.31% | 16.35% | 1.9123 | 15.09% |
| ≥60 years | 56 | 9.21% | 52 | 92.86% | 18.70% | - | - |
| Male | 32 | 5.26% | 30 | 93.75% | 9.12% | 1.7338 | 8.55% |
| Female | 24 | 3.95% | 22 | 91.67% | 9.58% | 2.4253 | 8.78% |
| Total | 608 | 100.00% | 465 | 76.48% (Crude) | 100.00% | - | 81.466% (Adjusted) |

**Note:** Post-sample adjustment was conducted using the Inverse Probability Weighting (IPW) method. The IPW Weight ($W_{i}$) for individuals in each stratum was calculated as: $W_{i}=\frac{\text{2020 Chinese Census Proportion}}{\text{Sample Proportion}}$. The adjusted overall seroprevalence is mathematically equivalent to the sum of the products of the sample seroprevalence and the census proportion for each stratum.
